# A new analytical model for the response curve in megavoltage photon beams of the radiochromic EBT3 films measured with flatbed scanners

**DOI:** 10.1002/acm2.13654

**Published:** 2022-05-17

**Authors:** César Rodríguez, Diego García‐Pinto, Luis Carlos Martínez, Alfonso López‐Fernández

**Affiliations:** ^1^ Medical Physics Radiology Department Complutense University Madrid Spain; ^2^ Medical Physics and Radiation Protection Service Fuenlabrada University Hospital Fuenlabrada Spain; ^3^ Medical Physics and Radiation Protection Service Doce de Octubre University Hospital Madrid Spain

**Keywords:** calibration uncertainty, EBT3, film dosimetry, radiochromic film, response curve

## Abstract

**Purpose:**

The aim of this work is to study a new analytical model which describes the dose–response curve in megavoltage photon beams of the radiochromic EBT3 film measured with two commercially available flatbed scanners. This model takes into account the different increase of the number of two types of absorbents in the film with absorbed dose and it allows to identify parameters that depend on the flatbed scanner and the film model, and parameters that exclusively depend on the production lot. In addition, the new model is also compared with other models commonly used in the literature in terms of its performance in reducing systematic calibration uncertainties.

**Methods and materials:**

The new analytical model consists on a linear combination of two saturating exponential functions for every color channel. The exponents modeling the growing of each kind of absorbent are film model and scanner model‐dependent, but they do not depend on the manufacturing lot. The proposed model considers the different dose kinetics of each absorbent and the apparent effective behavior of one of the absorbents in the red color channel of the scanner.

The dose–response curve has been measured using EBT3 films, a percentage depth dose (PDD) calibration method in a dose range between 0.5 and 25 Gy, and two flatbed scanners: a Microtek 1000 XL and an EPSON 11000 XL. The PDD calibration method allows to obtain a dense collection of calibration points which have been fitted to the proposed response curve model and to other published models. The fit residuals were used to evaluate the performance of each model compared with the new analytical model.

**Results:**

The model presented here does not introduce any systematic deviations up to the degree of accuracy reached in this work. The residual distribution is normally shaped and with lower variance than the distributions of the other published models. The model separates the parameters reflecting specific characteristics of the dosimetry system from the linear parameters which depend only on the production lot and are related to the relative abundance of each type of absorbent. The calibration uncertainty is reduced by a mean factor of two by using this model compared with the other studied models.

**Conclusions:**

The proposed model reduces the calibration uncertainty related to systematic deviations introduced by the response curve. In addition, it separates parameters depending on the flatbed scanner and the film model from those depending on the production lot exclusively and therefore provides a better characterization of the dosimetry system and increases its reliability.

## 1 INTRODUCTION

Radiochromic films are widely used as detectors to measure spatial distributions of absorbed dose. Their composition based on light elements, low energy dependence, near biological tissue equivalence, and high spatial resolution make them suitable detectors for advanced techniques in radiotherapy. Their measurement principle is based on the optical density change measured by a flatbed scanner.^[^ ^1^, ^2^ ^]^ The accuracy of the measurement depends on several aspects including: film handling, processing protocol, and calibration methods.^[^ ^3^, ^4^ ^]^

The curve relating the digital signal produced by the scanner, as a measurement of the light attenuation through the film, and the absorbed dose is commonly referred as the dose–response curve. This relationship is not linear and several phenomenological models have been proposed to describe it. Various analytical models have been used: polynomials,^[^ ^5^ ^]^ rational functions,^[^ ^6^ ^]^ a linear relation combined with a power function to account for the nonlinear response,^[^ ^7^ ^]^ exponentials,^[^ ^8^ ^]^ or more complex parameterizations.^[^ ^9^, ^10^, ^11^ ^]^

To measure the dose–response curve, multiple film pieces are usually irradiated inside a reference field with the film positioned perpendicular to the beam central axis.^[^ ^3^ ^]^ Alternatively, a PDD calibration method^[^ ^12^ ^]^ can be used.

The absorbed dose by a radiochromic film, induces a polymerization reaction in the monomer crystals forming the emulsion layer of the film, which alters their spectral light attenuation properties. The dose–response curve, as measured by the scanner, condenses this spectral information in one or three color bands. Several authors^[^ ^13^, ^14^, ^15^ ^]^ have determined the attenuation due to the film active layer as a spectral property. Devic et al.^[^ ^16^ ^]^ first showed that it was possible to describe the spectral attenuation combining several Lorentzian functions. Callens et al.^[^ ^15^ ^]^ established a model linking polymer conjugation length, energies of electronic and vibronic transitions, and polymer color phases. The absorption spectroscopy measurements are adequately described by this model.

A limitation of the previous studies is that the measurement conditions of spectral absorption could be different from those used to measure light attenuation in flatbed scanners. Spectral absorption is usually determined employing monochromatic light while the flatbed scanner uses broadband emission and detection, including some light scattering and wavelength shifting phenomena. Callens et al.^[^ ^17^ ^]^ and León‐Marroquín et al.^[^ ^18^ ^]^ have measured spectral absorptions under broadband conditions.

The aim of this work is to obtain, using a PDD calibration method, dose–response curve data that allow us to study the behavior of an analytical model which describes the dose–response curve of two commercially available flatbed scanners in a dose range between 0.5 and 25 Gy. The model is based on the increase with dose of the number of two types of absorbents, each one with its own kinetics. This model enables to separate the parameters depending on the flatbed scanner and the film model from those depending on the production lot exclusively. In addition, the new model is compared with other models commonly used in the literature in terms of its performance in reducing systematic calibration uncertainties.

## 2 METHODS AND MATERIALS

### 2.1 Dose–response curve model

The emulsion layer of the EBT2 and EBT3 radiochromic film models is composed of LiPCDA microcrystals dispersed in a water soluble material. The microcrystal constituents are diacetylenes monomers which under exposure to an external agent, such as heat, UV rays or ionizing radiation, undergo chain polymerization. The backbone of the polymer can be arranged according to different geometrical conformations,^[^ ^19^, ^20^ ^]^ giving rise to two detectable polymer color phases,^[^ ^15^ ^]^ each one with a characteristic torsion angle between two consecutive repeated linked units.^[^ ^21^, ^22^ ^]^

As the two polymer color phases exhibit different optical absorption properties, ionizing radiation induces the growth of two types of absorbents in the emulsion layer.^[^ ^15^, ^17^ ^]^ The absorbents are named after their predominant absorption color band. They are commonly referred as the red and blue phases.^[^ ^15^ ^]^ The total light absorption in the film results from the combined action of the two absorbents. The absorbent density increases with absorbed dose, but the dose dependence is different for each color phase which results in a complex relationship between dose and light absorption.^[^ ^17^, ^18^ ^]^

Let $\rho_{r}$ and $\rho_{b}$ be the red and blue absorbent density, number of absorption centers per unit of volume, for a given dose *D*. Let $\sigma_{r}$ and $\sigma_{b}$ be the red and blue effective light extinction cross sections per absorbent for a broadband light intensity *I*. Then, the relative differential reduction in light intensity −dI/I of a light beam crossing a differential volume of emulsion layer with unit area and thickness dx is equal to the total cross section of centers.^[^ ^17^ ^]^

(1) $$\frac{dI}{I}=-\left(\sigma_{r}\rho_{r}+\sigma_{b}\rho_{b}\right)\;dx.$$

Solving the differential equation (1) results the light intensity *I* that emerges from a thickness *t* of emulsion layer

(2) $$I=I_{0}\;e^{-(\sigma_{r}\rho_{r}+\sigma_{b}\rho_{b})\;t},$$

where *I*_0_ is the light intensity striking the emulsion layer.

The optical density *d* is defined^[^ ^6^, ^17^ ^]^

(3) $$d=\log_{10}\frac{I_{0}}{I}.$$

Combining Equations (2) and (3) results

(4) $$d=\left(\sigma_{r}\rho_{r}+\sigma_{b}\rho_{b}\right)\;t\;\log_{10}e=d_{r}+d_{b},$$

so the total optical density is the sum of the optical densities of two absorbent species.

For the color phase *p*, the number of centers per unit area $\rho_{p}\cdot t$ is a function of absorbed dose. Let us consider there is a number $n_{p}$ of lithium pentacosa‐10,12‐diynoate (LiPCDA) monomers per unit of volume laid out in such a way that they generate polymers of the phase *p*.^[^ ^15^ ^]^ After being irradiated by an absorbed dose dD, the number of polymers formed will be proportional to $n_{p}$ and an interaction probability $\kappa_{p}$. On average, every polyPCDA macromolecule will be a chain of $n_{m}$ LiPCDA monomers, being $n_{m}$ the mean conjugation length. The decrease in the number of monomers that are not yet polymerized will be

(5) $$dn_{p}=-n_{m}\;\kappa_{p}\;n_{p}\;dD.$$

Solving this differential equation results in the remaining number of not yet polymerized monomers for the *p* color phase in the emulsion layer

(6) $$n_{p}\left(D\right)=n_{0,p}\;e^{-\kappa_{p}n_{m}D},$$

where $n_{0,p}$ is the initial number of monomers per unit of volume arranged to become polymers of the phase *p*. The sum of free and conjugated monomers have to be equal to $n_{0,p}$. Taking into account $\rho_{p}\left(D\right)$, the density of formed polymers, then

(7) $$\begin{array}{c} n_{0,p}=n_{p}\left(D\right)+\rho_{p}\left(D\right)\;n_{m}=n_{0,p}\;e^{-\kappa_{p}n_{m}D}+\rho_{p}\left(D\right)\;n_{m}, \end{array}$$

from which it follows that the number of polymers formed belonging to phase *p* per unit volume as function of the dose will be

(8) $$\rho_{p}\left(D\right)=\frac{n_{0,p}}{n_{m}}\;\left(1-e^{-\kappa_{p}n_{m}D}\right).$$

Combining Equations (4) and (8) results in

(9) $$\begin{array}{ccc} d & = & t\;\left(\log_{10}e\right)\;\frac{n_{0,r}}{n_{m}}\;\sigma_{r}\;\left(1-e^{-\kappa_{r}n_{m}D}\right)\\ & & +\;t\;\left(\log_{10}e\right)\;\frac{n_{0,b}}{n_{m}}\;\sigma_{b}\;\left(1-e^{-\kappa_{b}n_{m}D}\right). \end{array}$$

The constants $n_{m}$, $\kappa_{r}$, and $\kappa_{b}$ are characteristics of the emulsion layer material and they do not depend on the production lot. Following the determinations from Callens et al.,^[^ ^15^ ^]^ $n_{m}$ is not dependent on the polymer color phase either. The other constants *t*, $n_{0,r}$, $n_{0,b}$ can change with the production lot. Since $\sigma_{r}$ and $\sigma_{b}$ are broadband effective cross sections, they depend on the spectrum of the light used to measure *d*. Furthermore, the measurements from Callens et al.^[^ ^17^ ^]^ and León‐Marroquín et al.^[^ ^18^ ^]^ have shown that the absorbance curves of the active layer are different under monochromatic and broadband conditions, and that the difference between them increases with absorbed dose. In that sense $\sigma_{r}$ and $\sigma_{b}$ should be expressed as dose functions. Unfortunately, the determination of the dose dependence of each $\sigma_{p}$ is a complex task, even analyzing the absorbance curves determined under broadband conditions. The specific characteristics of the device used to measure broadband absorbance curves affect their value. Besides that the effect of the $\sigma_{p}$ dose dependence always appears mixed in with the dose variation of the number of polymers in these curves.

Analyzing the integral absorbance in a dose range from 0.5 to 5 Gy using spectral absorbance curves measured under monochromatic conditions, in a previous article^[^ ^23^ ^]^ we proposed that the optical density as measured by a flatbed scanner can be expressed as

(10) $$d=\phi_{r}\;\left(1-e^{-k_{r}D}\right)+\phi_{b}\;\left(1-e^{-k_{b}D}\right),$$

where the saturation exponents of the color phases, $k_{r}$ and $k_{b}$, were characteristics of the emulsion layer and were considered not dependent on the scanner color channel nor the production lot. The parameters $\phi_{r}$ and $\phi_{b}$ were related to the relative abundance of each polymer color phase and proved to be color channel and production lot dependent in the dose range considered. Identifying terms in Equations (9) and (10), the production lot dependency of $\phi_{p}$ comes from the active layer thickness *t* and the initial number of specific precursors $n_{0,p}$. The value of each $\phi_{p}$ depends on the color channel because light attenuation is measured in a different wavelength band in which the absorption peaks of each polymer phase expresses in a different amount. Each color phase saturates with the same rate, independent of the color channel, as long as its contribution to the total optical density *d* is proportional to the polymer number, which it is shown to be an accurate approximation up to 5 Gy.^[^ ^23^ ^]^

The absorbance measurement under broadband conditions published by Callens et al.^[^ ^17^ ^]^ and by León‐Marroquín et al.,^[^ ^18^ ^]^ both covering a dose range up to 50 Gy, seem to indicate that the values of the absorbance corresponding to wavelengths in the red band behaves differently than the values in the green and blue bands. The red band is dominated by the main absorption peak of the red phase of the polymer. In that sense, Equation (10) will be no longer valid when applied to a wider dose range under broadband conditions, such as those of flatbed scanners. A plausible semiempirical model is to modify Equation (9) considering that for wavelengths around the main absorption peak of the red color phase, the $\sigma_{r}$ dose dependence can be reflected in a different effective number of polymers, that is, $\sigma_{r}$ and $\sigma_{b}$ will be considered constants although their values will depend on the wavelength band, and $\kappa_{r}$ will be different if determined in the red wavelength band than if it were determined in the green or blue wavelength bands.

If we define $d_{R}=\log_{10}2^{16}/R$, $d_{G}=\log_{10}2^{16}/G$, $d_{B}=\log_{10}2^{16}/B$ as the optical densities produced by the exposed emulsion layer and measured by a flatbed scanner in the three color bands with 16 bit depth digital signals *R*, *G*, and *B*, our hypothesis is that the dose–response curves can be expressed as

(11) $$\begin{array}{cc} d_{R} & =\phi_{r,R}\;\left(1-e^{-k_{r,R}D}\right)+\phi_{b,R}\;\left(1-e^{-k_{b}D}\right),\\ d_{G} & =\phi_{r,G}\;\left(1-e^{-k_{r,GB}D}\right)+\phi_{b,G}\;\left(1-e^{-k_{b}D}\right),\\ d_{B} & =\phi_{r,B}\;\left(1-e^{-k_{r,GB}D}\right)+\phi_{b,B}\;\left(1-e^{-k_{b}D}\right). \end{array}$$

Equations (11) reflect the common saturation behavior of the blue phase in the three color channels, and of the red phase in the green and blue channel. However, there is a specific parameter $k_{r,R}$ for the red color phase contribution to the red channel, due to the different behavior of the main absorption peak of this color phase. According to Equation (9), these parameters are related to optical properties of the emulsion layer material. They should depend on the film model and the flatbed scanner, but they should not vary by changing the production lot. The parameters ϕ for every color phase and color channel should depend on the film model, flatbed scanner and production lot as they incorporate the parameters *t*, $n_{0,r}$, and $n_{0,b}$ whose values are affected by uncertainties during the manufacturing process of the film.

### 2.2 Dose–response curve measurement

To measure the dose–response curve EBT3, films belonging to the production lot 06191802 were used. To manipulate the films, the recommendations given in Niroomand‐Rad et al.^[^ ^3^ ^]^ were followed. The films were cut into rectangular pieces, 5 cm width, 16 cm length. Care was taken to maintain the orientation of the film. A portrait orientation, relative to the uncut film, was chosen. The films were irradiated in a Siemens Artiste linear accelerator using photons with a nominal energy of 6 MV. The accelerator is subject to a quality control program. The dose output is well established and traceable. The dose output repeatability in the time taken for completing the PDD protocol is better than 1%. Each film piece was placed inside a cubic phantom of RW3 material. The geometry of the irradiation was with the plane of the film piece and its long side set parallel to the axis of the radiation beam.

The dose inside the phantom was calculated by means of an Acuros XB algorithm, validated under the same conditions using an ionizing chamber. The differences in the absorbed dose distribution alongside the depth axis determined by the Acuros XB algorithm and the ionizing chamber measurements were less than 0.2%.

A useful area inside the phantom was defined to avoid edge effects in the film. The irradiation conditions were set up to get a ratio between maximum and minimum in the percentage depth dose of two inside the useful area. To cover a wider dose range, several irradiation series were defined with different monitor units. Their values were set to get maximum absorbed doses of 1, 1.5, 2, 3, 4, 6, 8, 12, 16, and 25 Gy. In this way, the covered dose range went from 0.5 to 25 Gy and the range from 0.75 to 16 Gy is measured in two different series. To minimize the uncertainty coming from the lack of uniformity in the film, five different film pieces were irradiated in each series.

The films were scanned 24 h after the irradiation. Two different A3 flatbed scanners were used in this study, a Microtek ScanMaker 1000 XL and an EPSON Expression 11000 XL. The scanning protocol was the same in both scanners. The films were positioned in the center of the scanning plate, with the axis containing the depth dose distribution oriented perpendicular to the scanning lamp so the lateral artifact is negligible. The scanner was switched on for several hours and its lamp was lit for at least 15 min to stabilize its temperature before scanning. The scanners were set in transmission mode. The scanned image was 72 dpi in spatial resolution and the digital signal was 48 bits in depth, 16 bits for each color channel. Any other additional correction by the scanner was switched off. Five consecutive scans of each calibration sample were taken and the average image was analyzed. To ensure the stability of the digital signal of the scanner, three rectangular film pieces, 1 cm width, 3 cm length, irradiated uniformly with absorbed doses of 2, 8, and 16 Gy were scanned together with the calibration series films. The mean digital signal of each stability piece was recorded. Three reference stability values were defined as the average of all mean digital signals belonging to same dose level. Each individual scan was corrected linearly by the ratios of these references to its current mean stability values. To reduce scanning noise, a nonlocal means algorithm was applied.

Contiguous square areas of 3 × 3 mm^2^ were defined in the direction of the long axis of the calibration film pieces. Their mean digital signals were converted to optical densities using the definition given in the previous section. Optical densities in these areas were assigned to their absorbed doses as calculated by the Acuros XB algorithm.

The relationship between absorbed dose and optical density for every color channel was fitted to the proposed response curve model, Equation (11). The fit was made using a least square method by means of a Levenberg–Marquardt algorithm. An implementation written in the python programming language in a module called lmfit was used. The parameter $k_{r,GB}$ was shared by the green and blue channels and the parameter $k_{b}$ was shared by all the three color channels. An additional linear parameter was fitted in order to determine the background optical density for every color channel, and then it was subtracted as we are analyzing the response of the emulsion layer.

The residuals of the curve fit was evaluated to identify systematic deviations in the response curve model. The value of χ^2^ normalized by the number of the degrees of freedom for every fit was calculated considering the optical density measurement uncertainties. Probability plots were used to study the residual normality.

### 2.3 Comparison with other dose–response curve models

The same calibration data were fitted to other published calibration models using the same algorithm. Table 1 presents a summary of the models considered in this work. The systematic deviations produced by these models were identified by inspecting each curve fit residuals. The χ^2^ normalized by the number of degrees of freedom using the same uncertainties considered in the previous section was calculated as well for every model, and they were used to evaluate the relative performance of each model compared with the proposed model. The residual normality was studied by means of normal probability plots.

**TABLE 1:** Different published models relating optical density *d* and absorbed dose *D*

ID	Mathematical expression	Reference
Rational	$d=-\log_{10}\frac{a+bD}{c+D}$	Micke et al.^[^ ^6^ ^]^
Polynomial	$d=a+b\;D+c\;D^{2}++d\;D^{3}$	Borca et al.^[^ ^5^ ^]^
Linear power	$D=a\;d+b\;d^{c}$	Devic et al.^[^ ^7^ ^]^
Exponential	$d=a-b\;e^{-c\;D}$	Poppinga et al.^[^ ^8^ ^]^
Tamponi	$\varepsilon \left(R\right)=\frac{\log[1+\left(A_{c}-1\right)\;R]}{\log(A_{c})}$ $A_{c}=1+h_{c}\;D_{r},\;R=\frac{D}{D_{r}},\;\varepsilon \left(R\right)=\frac{d(D)}{d(D_{r})}$	Tamponi et al.^[^ ^9^ ^]^
SHGD	$d\left(D\right)=c\left(1-\frac{a^{b}}{{\left(a+D\right)}^{b}}\right)$	del Moral et al.^[^ ^10^ ^]^

*Note*. The identification SHGD stands for the Single Hit Gamma Distributed model.

### 2.4 Application to different production lots

To verify the expected independence of the saturation exponents with the production lot, the calibration data from three different lots (10091501, 12051602, and 06191801) were retrospectively analyzed. In this case, calibration irradiations were made using square pieces of 3 × 3 cm^2^ placed perpendicular to the radiation beam axis inside a cubic phantom of RW3 material. Each film piece was irradiated with a uniform dose of 0, 0.5, 0.75, 1, 1.25, 1.5, 2, 3, 4, 5, 7, and 9 Gy. This calibration procedure was repeated for the lot 06191802 as well.

For every production lot, the film pieces were scanned all at the same time, using the Microtek ScanMaker 1000 XL, 24 h after irradiation. The scanning protocol was the same that the one previously explained. In this case, the average digital signal of every calibration piece was converted to optical density and related to the absorbed dose.

The response curve was fitted using a least square method allowing all the ϕ parameters to vary, but using the $k_{r,R}$, $k_{r,GB}$, and $k_{b}$ determined using the production lot 06191802 and the PDD calibration procedure.

## 3 RESULTS

**FIGURE 1:**
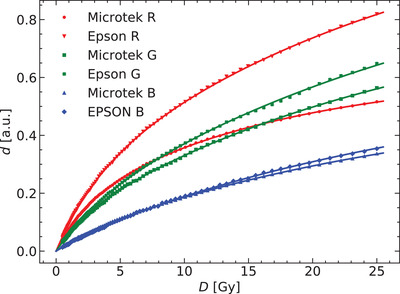
Obtained response curves for the two flatbed scanner models and for the three color channels. Continuous lines are the fit of the measured values to the proposed model

In Figure 1, the optical densities corresponding to a dose range between 0.5 and 25 Gy and measured using the two flatbed scanners are shown. Note the different *d* values depending on the scanner and on the color channel. The figure also shows the fit of these measured values to the Equations (11).

**FIGURE 2:**
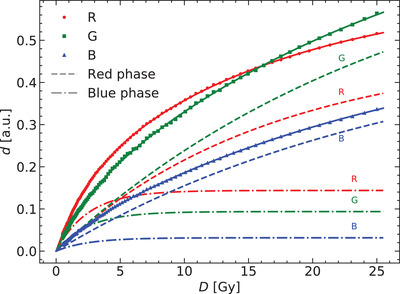
Breakdown of contributions by polymer color phases to the response curve for each color channel of the Microtek 1000 XL scanner

Figure 2 shows how each polymer color phase contributes to the total optical density. Data shown belong to the scanner Microtek. The blue color phase saturates for doses above 10 Gy, so the variation in the optical density is only due to the variation of the red color phase. Note as well the different dose dependence of the red phase in the red channel compared to its behavior in the green and blue channels.

**FIGURE 3:**
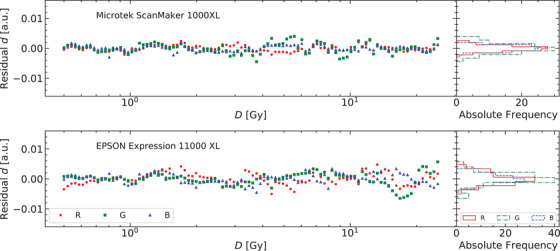
Residuals for the two scanners and the three color channels fitted to the proposed model plotted versus absorbed dose values and histogrammed

**FIGURE 4:**
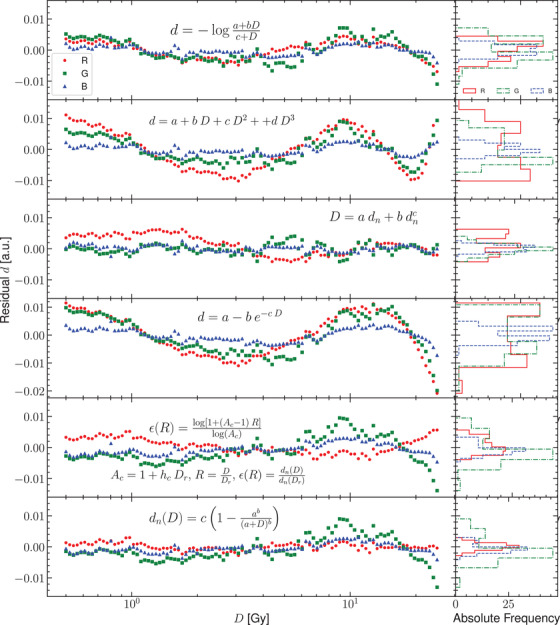
Comparison of the residuals for the Microtek 1000 XL flatbed scanner fitted to different published analytical models

**FIGURE 5:**
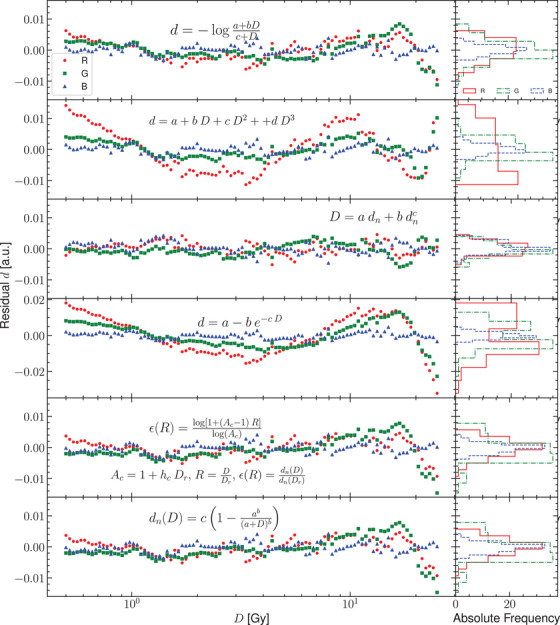
Comparison of the residuals for the EPSON 11000XL flatbed scanner fitted to different published analytical models

**FIGURE 6:**
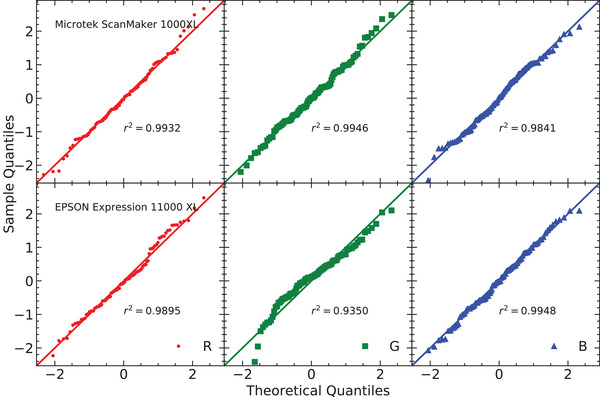
Normal probability plots of the residuals for the Microtek and EPSON scanners when using the response curve proposed in this work. The term *r* ^2^ denotes the determination coefficient obtained considering these residual should be normally distributed

**FIGURE 7:**
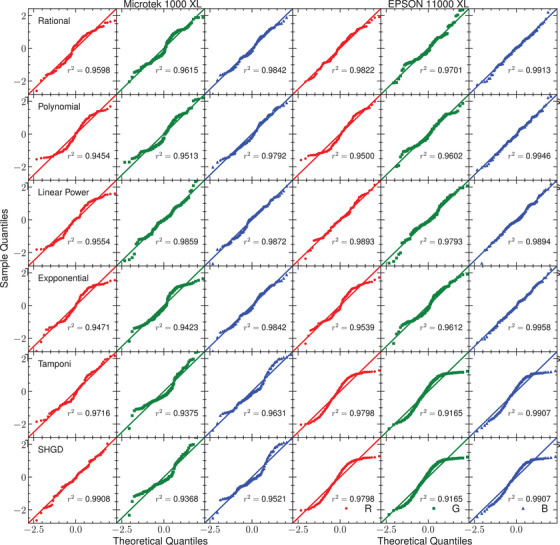
Normal probability plots of the residuals for the Microtek and EPSON scanners when using other published response curve models. The coefficient *r* ^2^ has the same meaning as in Figure 6

**FIGURE 8:**
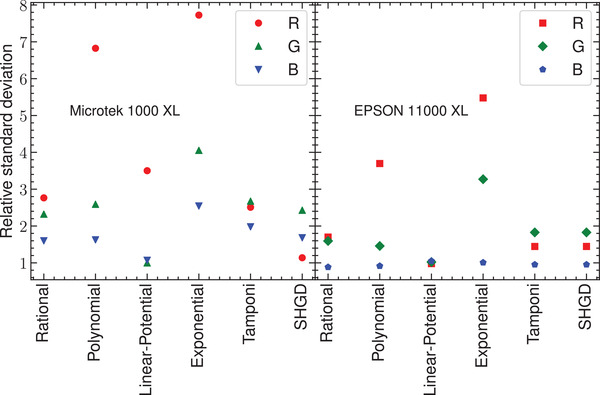
Standard deviations of the residuals for each color channel and for each analytical model divided by the standard deviation of the proposed model for each color channel and scanner model

In Figure 3, the residuals of the response curve for the two flatbed scanners and their corresponding histograms are presented. Figures 4 and 5 show the residuals for other published analytical models for the scanners Microtek and Epson, respectively. The normal probability plots of the residuals for both scanner models obtained using the response curve proposed in this work are shown in Figure 6. Figure 7 shows the normal probability plots for the other published response curves and scanner models. In Figure 8, the standard deviation of the residual distribution is quantified in relative terms to the corresponding standard deviations of the proposed model. Table 2 lists the χ^2^ normalized by the number of degrees of freedom of each fit for every response curve model, color channel, and scanner model.

**TABLE 2:** χ^2^ normalized by the degrees of freedom for the different studied response curve models

	Microtek 1000 XL			EPSON 11000 XL		
	R	G	B	R	G	B
This work	0.885	1.479	0.842	0.962	0.998	0.812
Rational	4.338	8.777	1.300	2.769	2.811	0.532
Polynomial	26.704	11.027	1.356	13.235	2.378	0.575
Linear power	2.466	2.723	0.411	1.202	1.930	0.497
Exponential	33.863	26.759	3.287	28.758	11.832	0.690
Tamponi	200.949	629.059	450.093	382.085	414.049	259.664
SHGD	203.054	635.610	454.774	386.079	418.349	262.363

*Note*. An uncertainty of 0.0013 for the Microtek scanner and an uncertainty of 0.002 in the optical density measurement were considered.

**FIGURE 9:**
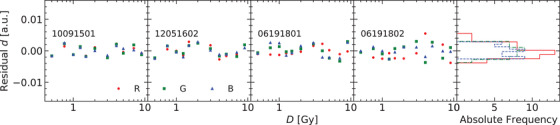
Residuals of the response curve for four different production lots when fitted allowing just the ϕ parameters to vary, but using the $k_{r,R}$, $k_{r,GB}$, and $k_{b}$ determined using the PDD calibration procedure

As the exponential terms in Equations (11) are considered independent of the production lot, the values determined using the PDD calibration method can be used to fit the response curve of other lots. Figure 9 shows the fit residuals for four different production lots obtained by fitting just the linear coefficients in Equations (11).

## 4 DISCUSSION

As shown in Figure 1, the PDD calibration method allows to characterize the dose–response curve with a large number of data points, higher than the number usually available using patches positioned perpendicular to the beam axis. This dense calibration best characterizes the dose variation of the optical density, allowing to distinguish the subtle differences between different analytical models.

Figure 1 graphically shows the variation of the optical density produced by different scanners while measuring the same sample. These differences have already been reported by several authors,^[^ ^3^, ^24^ ^]^ and originate in the design of the optical elements of the scanner.^[^ ^25^, ^26^ ^]^

Equation (11) allows us to quantify the optical density due to each color phase. Up to 10 Gy both color phases contribute to the variation of the optical density with absorbed dose, but for higher dose values the measured optical density variation is mainly due to the red phase. Callens et al.^[^ ^15^ ^]^ developed a model, based on theoretical and experimental basis,^[^ ^21^, ^22^, ^27^, ^28^, ^29^ ^]^ to determine the contribution of each color phase to the optical density, and the mean conjugation length of the polymer chains in the films. This study showed that the polymer phases have different dose‐dependent reaction kinetics. Using the spectral absorbance model proposed by Callens et al., a sensitometry model for EBT film models and flatbed scanners was developed^[^ ^23^ ^]^ based on the assumption that the color phase optical density dose dependence is related to the variation with dose of its integral absorbance. This study assumed as well that the variation of the optical density with absorbed dose for each color phase is color channel‐independent, that is, this variation is proportional to the actual number of polymers. The dose range considered in the study was up to 5 Gy. According to the spectral absorbance measurements of the emulsion layer of EBT3 films under broadband conditions,^[^ ^17^, ^18^ ^]^ similar to those of flatbed scanners, the absorbance curves present broader absorption peaks compared with the monochromatic absorbance. The relative intensities of the peaks are different as well and it is no longer acceptable to assume that each color phase optical density is proportional to the polymer number. The higher the dose, the more marked the difference between monochromatic and broadband absorbance curves. The difference is notable for the main absorption peak corresponding to the transition from the ground level to the first excited electronic level of the blue phase, λ= 635 nm. As this peak is in the red color channel of the scanner, the optical density dose dependence in this channel is different to those in the green and blue channels. In spite of the absorption peak broadening being due to optical effects, it can be interpreted by the scanner as an effective absorption center number variation, which allows us to formulate Equations (11). These equations expresses through the different saturation rates $k_{r}$ the specific characteristics of the digital scanner optical systems considered in this study.

The residuals shown in Figure 3 help us to evaluate the accuracy of the proposed model to describe the response curve. Together with Figures 4 and 5 they allow to identify systematic deviations introduced by each analytical model. Figure 3 shows residuals of equal magnitude for all color channels and both scanners. Figure 6 shows that the distributions of residuals are mostly normally shaped, which suggests that the model is not introducing any systematic errors or, at least, the systematic errors are not bigger than random errors. The bigger deviation from normality occurs for the green channel of the EPSON scanner and it seems to correspond to doses higher than 10 Gy. There are not strong correlations between the residuals corresponding to both scanners supporting the fact that most of the variation is due to random fluctuations in the response curve data. The proposed model describes slightly better the response curve for the Microtek flatbed scanner than for the EPSON scanner, with regard to the red and blue color channels. The Microtek green channel presents residuals of the same order than the three color channels of the EPSON scanner. The green channel of this scanner may be increasing its residual values for doses higher than 10 Gy.

Figures 4 and 5 show the distributions of residuals for the other models considered that have in general higher variances (see Figure 8) and are mostly not normally shaped (see Figure 7) probably indicating the introduction of systematic deviations. The values of χ^2^ listed in Table 2 support this result. The variance reduction implies that calibration uncertainties are reduced by a mean factor of two when using the proposed model. An important difference between this model and the analytical models used in these figures is that Equations (11) provide a global response curve model for the scanner, which describes the different behavior of the red color channel and shares between the green and blue channels the parameters describing the dose variation. The analytical models used in Figures 4 and 5 share the same functional form for the three channels with independent parameters for each one. when using these analytical models, it could be a better choice to select the analytical model which better describes the physical behavior of each channel.

It is interesting to note that some of these analytical models, despite having different functional forms, produce quite similar results in the end. It is the case of Tamponi and SHGD models, specially for the EPSON scanner. For the Microtek scanner, there are substantial differences only for doses higher than 15 Gy.

One of the characteristics of Equations (11) is that they allow us to separate parameters that depend on the film production lot from parameters depending on the scanner and film model. Figure 9 shows the residuals of applying this approach to four different production lots. Only the linear parameters describing the different proportion of each polymer color phase have been fitted. No bias or systematic deviation is observed in the four production lots. According to this model, once the parameters related to the saturation rates are determined, which are the ones depending on the scanner and film model, the linear parameters can be established by using a simple calibration process that could even be reduced to just two calibration dose levels. The saturation rates are expected to be common parameters for scanners with the same optical system design and using the same film model, which would allow them to be externally provided, although more work has to be done to test this issue.

## 5 CONCLUSIONS

A response curve model has been proposed which is based on the increase with dose of the number of two types of absorbents, each one with its own kinetics. The model takes into account as well the apparent different effective behavior of one of the absorbents when it is measured under broadband conditions.

To study the dose–response curve, a PDD calibration method has been used. By means of this method is possible to efficiently produce a dense collection of pair calibrations points, relating absorbed dose and optical density which allows us to determine the parameters of the dose–response model and to identify systematic deviations introduced by the analytical functional form.

The proposed response curve model does not introduce systematic deviations up to the degree of accuracy reached in this work. One additional strength of the model is that it enables to separate the parameters depending on the flatbed scanner and the film model from those depending on the production lot exclusively. The first ones are related to the specific characteristics of the dosimetry system. The lot dependent ones, which reflect the relative abundance of each type of absorbent, are linear parameters so they are easily determinable facilitating the calibration process. Using this model the uncertainties related to the fitting process in the calibration are, on average, halved compared with other published models.

## CONFLICT OF INTEREST

The authors have no conflict of interest to disclose.

## AUTHOR CONTRIBUTIONS

All listed authors contributed substantially to the study design and execution, and the drafting and reviewing of the manuscript. Each author approved the final submitted version of the manuscript.
